# Tissue- and temporal-specific roles of extracellular ATP on T cell metabolism and function

**DOI:** 10.1097/IN9.0000000000000025

**Published:** 2023-05-01

**Authors:** Igor Santiago-Carvalho, Alma Banuelos, Henrique Borges da Silva

**Affiliations:** 1 Department of Immunology, Mayo Clinic Arizona, Scottsdale, AZ, USA

**Keywords:** T cells, eATP receptors, tissue damage, P2RX7, T cell memory

## Abstract

The activation and function of T cells is fundamental for the control of infectious diseases and cancer, and conversely can mediate several autoimmune diseases. Among the signaling pathways leading to T cell activation and function, the sensing of extracellular adenosine triphosphate (eATP) has been recently appreciated as an important component. Through a plethora of purinergic receptors, most prominently P2RX7, eATP sensing can induce a wide variety of processes in T cells, such as proliferation, subset differentiation, survival, or cell death. The downstream roles of eATP sensing can vary according to (a) the T cell subset, (b) the tissue where T cells are, and (c) the time after antigen exposure. In this mini-review, we revisit the recent findings on how eATP signaling pathways regulate T-cell immune responses and posit important unanswered questions on this field.

## 1. Introduction

In response to infectious and noninfectious diseases, the release of several inflammatory mediators, either through lytic release or via active export by live cells, is a normal step. Among these mediators, we and several others have studied the effects of extracellular adenosine triphosphate (eATP) accumulation because of these disease states ^[1,2]^. ATP, typically, is found inside cells, being the quintessential “fuel” for energy-demanding intracellular processes ^[3]^. But in inflammatory situations (and at a lesser extent during homeostasis), eATP accumulates in the surrounding microenvironment ^[2]^. eATP, once present, can bind to many distinct membrane receptors – those are called purinergic receptors ^[1,4]^. P2 purinergic receptors that bind eATP can be either P2RX ion channels (that lead to Ca^2+^ influx) or P2RY G-protein coupled receptors that drive, among other signals, cyclic adenosine monophosphate (cAMP)-and/or Ca^2+^-induced signaling pathways ^[4]^. Conversely, the action of several ectoATPases limits the availability of eATP in a temporal and context-dependent way ^[4]^. In the last few years, a large body of research has been conducted to understand how sensing of eATP by these receptors regulates T-cell immune responses. In this mini-review, we revisit some of these findings and enlist important existing knowledge gaps. Despite recent evidence that unconventional T cells rely on purinergic signaling for their function ^[5,6]^, in this review we will focus on the recent findings about eATP signaling in CD4^+^ and CD8^+^ T cells. We mostly focus on how eATP sensing via P2RX receptors regulates T-cell responses.

## 2. The role of eATP receptors in T cell development and function

As crucial promoters of immunity to diseases, CD4^+^ and CD8^+^ T cells must recognize not only cognate antigen presented via Molecular Histocompatibility Complex (MHC) molecules ^[7]^, but also sense cues from the extracellular microenvironment. Signaling through eATP sensing is part of this: T cells express varied eATP receptors, and multiple aspects of their function and homeostasis rely on this ^[1,4]^. Depending on their subset or developmental stage, CD4^+^ and CD8^+^ T cells can express multiple eATP receptors. Among eATP receptors expressed in T cells, the ones with known functions are four: P2RX1, P2RX4, P2RX7, and P2RY11 ^[1,8]^. P2RX7, a unique low-affinity eATP receptor, is the one with the most well-described roles for T cell biology ^[1]^. P2RX7 has a consensual dual function in T cells, which seemingly depends on the intensity of the signal propagated: weak-to-intermediate signals induced transiently by moderate eATP concentrations (above 50 μM but below 1 nM) often promote T cell survival or function, while strong signals induced by too high eATP levels (>1 nM) or by covalent modifications of P2RX7 typically lead to T cell death due to the formation of large, nonspecific plasma membrane pores ^[1]^.

The development of T cells in the thymus is perhaps the best example of a situation where sensing of eATP is considered universally deleterious. The thymus is an organ where eATP accumulation can induce thymocyte death ^[9]^. A previous report has shown that expression of P2RX7 is negatively regulated by the activity of the histone deacetylase 3 (HDAC3); this ensures thymocytes will successfully go through thymic selection without undergoing cell death induced by too strong eATP signaling ^[10]^. By knocking out HDAC3, P2RX7 expression is promoted via the activity of retinoic acid-related orphan receptor γt (RORγt), and increased numbers of T cell precursors die ^[10]^. The low-to-negative P2RX7 expression in thymocytes was also found by our group (unpublished data).

Once in the periphery, antigen-inexperienced T cells typically re-circulate between blood, lymph, and secondary lymphoid organs ^[11]^. Four eATP receptors are known to be expressed in these cells: P2RX1, P2RX4, P2RX7, and (only in humans) P2RY11 ^[1,8]^. Another receptor, P2RX5, has reported expression in activated T cells ^[12,13]^ but its function is unknown. Whether sensing of basal eATP plays a role in these naïve T cells was not explored until recently. A recent work has shown that P2RX7 limits the homeostatic migration of naïve T cells, leading to retention within lymph nodes ^[14]^. A report published last year showed that preactivation of naïve T cells before T cell receptor (TCR)/MHC signaling, which is necessary for proper T cell priming ^[15]^, relies on basal mitochondrial-derived ATP ^[16]^ release via the Pannexin-1 channel and autocrine signaling via P2RX4, more precisely the formation of Ca^2+^ microdomains ^[17]^. This was the first report suggesting that sensing of eATP is not only necessary to amplify the activation of T cells after priming (more on this below) but may also lower the TCR signal strength threshold for full T cell activation. P2RX1 is possibly part of this network since it can be activated by even lower eATP concentrations if compared to P2RX4 ^[16]^. Previous studies also suggested a role for P2RX7 ^[18]^, although those were reported in Jurkat T cells, which are immortalized human cell lines – therefore, not necessarily reflecting what happens in primary T cells.

T cell eATP receptors play important roles after cognate antigen priming, both at early and late stages of activation. Both P2RX4 and P2RX7 promote the formation of Ca^2+^ microdomains in early activated T cells ^[17]^. These eATP-induced Ca^2+^ ultrastructures are important in the early propagation of the downstream signals needed for T cells to acquire the effector program and proliferate ^[17]^. This occurs as a continuation of the basal (ie, pre-priming) signals induced by Pannexin-1-exported eATP, and subsequent autocrine recognition by P2RX4 and P2RX7. This action is amplified after TCR priming, which increases the intensity of eATP signaling through these two receptors ^[17]^. P2RX4 and P2RX7 have also been suggested to amplify the global Ca^2+^ signaling at later stages of T cell priming ^[19–23]^, through induction of nuclear factor of activated T cells (NFAT) activation ^[21,22,24,25]^. As a result, it has been believed that P2RX4 or P2RX7 favors proper T-cell proliferation and cytokine production ^[16,21,22,26,27]^. These proposed roles are mainly based on in vitro findings with isolated human T cells or Jurkat T cell lines ^[21–23]^. In contrast to this notion, our group has shown that, in LCMV-infected mice, P2RX7 deficiency does not affect the proliferation and initial expansion of antigen-specific CD8^+^ T cells ^[28]^. Therefore, the importance of eATP receptors for the activation of T cells may depend on the antigen context, or on the T cell subset studied. More detailed assessments of in vivo mouse models and primary human T cells will be necessary to precisely pinpoint the role of eATP sensing here. An important difference between human and mouse T cells is their susceptibility to eATP-induced cell death. Human T cells are much more resistant to eATP-induced death ^[29]^, possibly due to the action of another eATP sensor, P2RY11, which inhibits the formation of P2RX7 pores ^[30,31]^. P2RY11 is not expressed in mice ^[8]^. Whether this could explain why P2RX7 has reported roles in initial T cell activation, yet no noticeable role in mice is a possible source of investigation.

After activation and the initial burst of clonal expansion, T cells still rely on eATP sensing for their differentiation into distinct functional subsets, mostly through P2RX7. For CD8^+^ T cells, P2RX7 is important to control the differentiation into memory subsets of different migratory characteristics, mostly through control of their metabolism and/or their susceptibility to eATP-induced cell death ^[28,32–35]^. We will further discuss this in the next sections. For CD4^+^ T cells, P2RX7 positively regulates their differentiation into T_H_1 and T_H_17 subsets ^[36,37]^. Conversely, P2RX7 negatively regulates the follicular helper (Tfh) subset ^[37–39]^ and seemingly restricts the regulatory T cell (Treg) subset ^[40]^. P2RX7 mostly induces Tfh cell death due to excessive eATP signaling. Tfh cells do not express CD39, an ectoATPase that metabolizes eATP and, therefore, limit excessive exposure of cells to eATP ^[37,39]^. Tregs are also believed to die through P2RX7 ^[40]^, but this subset typically expresses CD39. This apparent discrepancy is still not very well explained. A possibility could be enhanced exposure to nicotinamide adenine dinucleotide (NAD^+^). In mice, NAD^+^ can lead to covalent modifications of P2RX7 and irreversible pore formation, through the action of the ADP-ribosylase ART2.2 ^[29]^. However, Tregs can also express CD38 ^[41]^, a well-known NAD-cleaving enzyme. That leads back to our original conundrum: how can Tregs be susceptible to P2RX7-induced cell death even in the presence of enzymes that would be protective? Future studies will be needed to precisely define how P2RX7 regulate the Treg subset. As it will be discussed below, eATP sensing may regulate this subset through intracellular metabolic alterations.

## 3. Metabolic control of T cells through eATP receptors

One of the most well-known downstream results of eATP sensing is the regulation of T cell intracellular metabolism, which is fundamental to drive T cell function and homeostasis ^[1]^. Recently, this has been studied in more detail in CD8^+^ T cells. CD8^+^ T cells rely on successive metabolic adaptations to differentiate from naïve to effector to memory subsets ^[42]^. While naïve CD8^+^ T cells mostly rely on mitochondrial metabolism and oxidative phosphorylation for energy production, effector CD8^+^ T cells switch towards preferential usage of aerobic glycolysis for this ^[43–45]^. After antigen elimination and memory generation, a reversion to preferential mitochondrial-based energy production is crucial for the longevity of memory CD8^+^ T cells ^[46–48]^. P2RX7 has reported roles in both inducing mitochondrial metabolism and aerobic glycolysis ^[49,50]^ and can even locate at the mitochondria membrane ^[51]^. This makes it hard to imagine how this receptor could control CD8^+^ T cells toward effector or memory fates. Among effector CD8^+^ T cells, a portion of those were found to express high P2RX7 levels as early as 4 days after lymphocytic choriomeningitis virus (LCMV) infection, and these cells are marked for memory development ^[34]^. As a logical consequence, P2RX7-deficient CD8^+^ T cells significantly fail to develop into long-lived memory populations ^[28,35]^.

The impact of P2RX7 varies depending on the memory subset, and that follows its expression levels in each group. If divided based on migratory characteristics, memory CD8^+^ T cells can be roughly divided into four major subsets: central memory (T_CM_), effector memory (T_EM_), long-lived effector cells, and resident memory (T_RM_). T_CM_ and T_RM_ cells express higher levels of P2RX7, and ablation of this receptor preferentially affects these subsets ^[28,34,35]^. We recently found that P2RX7 promotes mitochondrial homeostasis and respiration in nascent memory CD8^+^ T cells ^[28]^, which is in turn regulated, at least partially, by P2RX7 inducing the adenosine monophosphate (AMP)-activated protein kinase (AMPK) pathway ^[28]^ and enhanced sensitivity to environmental transforming growth factor-beta (TGF-β) ^[34,35]^. Previous reports have shown a role for TGF-β in shutting down the mechanistic target of rapamycin (mTOR) pathway which leads to increased mitochondrial function – in Tregs ^[52]^. Our findings, however, suggest that sensing of eATP plays a role upstream of this interaction. How P2RX7 intracellularly regulates the TGF-β/mTOR/mitochondria axis, and whether this is acting together with AMPK, are unanswered questions that will help further understanding how this eATP receptor regulates CD8^+^ T cell metabolism and homeostasis. Another interesting finding is that P2RX7 is not only required for the development of T_CM_ and T_RM_ cells but also for their long-term survival ^[35]^. That precludes that P2RX7 must encounter eATP after infection and antigen is gone, in a time point where the lytic release of ATP is unlikely to be a major source of this nucleotide. Active release of ATP via transmembrane channels can also occur, and CD8^+^ T cells (and other T cell subsets) express a prominent ATP-exporting channel, Pannexin-1 ^[22,28,53,54]^. Whether Pannexin-1 is a provider of local eATP sources for already established memory CD8^+^ T cells, is still not understood and is a current subject of the investigator from our group and others. We had discussed these possible interactions in a previous mini-review ^[55]^.

CD4^+^ T cell effector subsets, as shown in the previous section, are regulated by P2RX7. Much of the modulatory role of this receptor may happen via metabolic control of these subsets. For instance, the negative regulation of Treg via P2RX7 could occur, instead of cell death, through P2RX7-induced mitochondrial damage induced by exaggerated signaling through this receptor ^[1]^ although this has not been formally tested. Conversely, T_H_17 cells depend on increased glycolytic activity, which could be promoted by preferential P2RX7 induction of glycolysis in these cells ^[56,57]^. In addition to direct control of mitochondrial metabolism versus glycolysis, P2RX7 could regulate CD4^+^ T cell fate choice and metabolism indirectly. P2RX7 can activate the Hypoxia-Inducible Factor 1 α (HIF1α) ^[50]^, which is an important positive regulator of glycolytic activity ^[58]^. HIF1α can block Treg differentiation through disruption of Foxp3 expression ^[59]^. In a similar direction, another regulatory T cell subset, type 1 regulatory cells (Tr1), is inhibited by P2RX7-induced HIF1α activity – in this case, because of disruption of the aryl hydrocarbon receptor, which is fundamental for the differentiation of this subset ^[60]^. If the P2RX7-HIF1α axis is required for overall CD4^+^ (and CD8^+^) T cell fate choice and metabolism, it still needs to be defined in future studies.

## 4. Does tissue localization and time matter?

**Figure 1.**
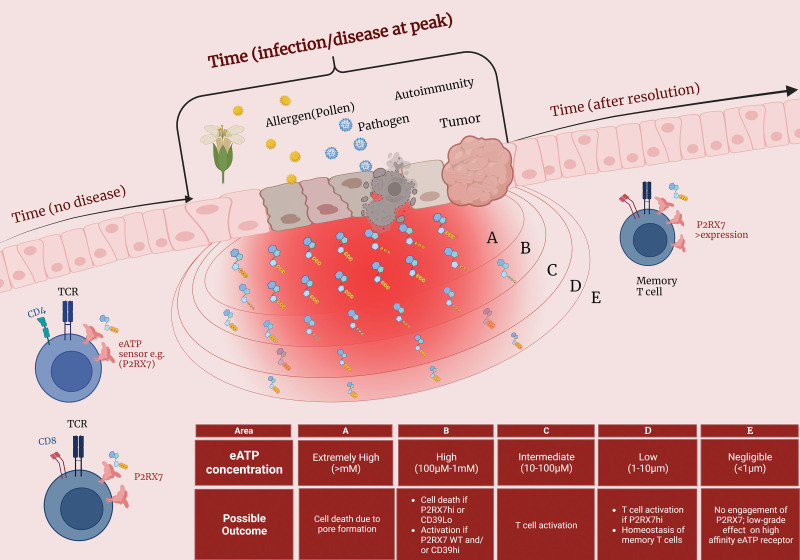
**Tissue localization and time-induced regulation of the role of eATP for T cell responses.** In this schematic figure, we postulate possible scenarios by which the spatial distribution of T cells, as well as time after disease induction, can modulate how eATP sensing occurs. With regards to tissue localization, we postulate that disease (allergy, infection, autoimmunity, or cancer) would likely induce focal points of eATP accumulation. From these areas, eATP levels would gradually decay with distance from these focal points. Because, for P2RX7 and other eATP sensors, the eATP concentration matters, the presence of T cells in each one of the “subareas” (exemplified with A-E letters) would define possible outcomes for eATP sensing. Simultaneously, time after disease induction will dictate the presence or absence of these focal points. In absence of disease, autocrine/paracrine eATP release (through Panx1 channels) is likely to be the dominant eATP source. In these circumstances, extremely high eATP levels that could induce T cell death are unlikely. eATP, extracellular adenosine triphosphate.

Sensing of eATP is, therefore, an inducer of multiple downstream effects in different conventional T cell subsets, and many of these effects are contrasting. These recent findings suggest that eATP receptors may promote distinct downstream metabolic pathways depending on factors external to the presence of these receptors. That can, as we stated before, be related to the expression of other receptors and enzymes on T cells themselves, such as CD39 or CD38. It is possible, however, that the surrounding microenvironment regulates how eATP sensing works in T cells. Two major variables could be relevant for this: tissue localization and time (**Figure 1**). With regards to location, it is important to remember that T cells, although all generated in the same organ (thymus), can be found virtually everywhere in the body. That is due to the ability of T cells to differentiate into tissue-resident populations ^[61]^. Many of the landmark findings on the role of eATP for immunity are based on the high levels of eATP found in tissues and tumors undergoing inflammation ^[2,62]^, as well as in the small intestine, where microbiota is an important eATP source ^[35,38]^. In T cells present in these organs, the sustained high levels of eATP often lead to increased susceptibility to cell death. This is the case for CD8^+^ T_RM_ cells in the small intestine and in other nonlymphoid tissues, where exposure to exaggerated NAD or eATP levels can induce their death ^[32,33]^. It is also true for intratumoral T cells in melanoma, which often show T_RM_ phenotypic traits ^[63]^ and are enriched inside tumors when P2RX7 is ablated ^[64]^. We have recently found that high levels of ATP guide the accumulation of influenza-specific CD4^+^ T cells in the lung parenchyma, determining the severity of the lung pathology (data not shown ^[65]^). However, expression of P2RX7 by these cells could be beneficial, given the right context – that is, exposure to low-to-intermediate eATP ^[35,66]^. Therefore, the tissue-dependent role of eATP receptors is likely to be also context-dependent.

In addition to tissue dependency, time is fundamental to regulate how eATP receptors influence T-cell responses. Usually, this pertains to time after antigen recognition, and two major parameters can change due to this: availability of eATP and expression of eATP receptors by T cells. The time changes in eATP levels reflect the levels of pathogen load and are a result of increased eATP release, which subsides after pathogen elimination ^[1]^. In parallel, T cells dynamically modulate the expression of different eATP receptors over time, especially the low-affinity P2RX7 receptor ^[32,35,37,39]^. The upstream regulation of P2RX7 is still under investigation for T cells, additional signals are probably needed for further differentiation ^[1,34]^. Nevertheless, P2RX7 may be partly induced by TCR stimulation, and effector T cells often display increased levels of this receptor if compared to naïve cells ^[35]^. Therefore, post-priming T cells would theoretically be more susceptible to cell death induced by higher concentrations of eATP, which increase concurrently. However, effector T cells not only survive during this period but expand and differentiate normally, even with higher P2RX7 expression ^[28,35,37]^. This contrast may be explained by tissue localization-dependent contexts: it is possible that expression of P2RX7 by effector T cells, which is heterogeneous, may favor the survival of cells that are not in direct contact with the epicenter of inflammatory areas. In other words, the successful establishment of P2RX7-expressing T cells into inflamed tissues may be dictated by their localization within areas of intermediate eATP levels, rather than within the inflammation center where eATP levels are high (**Figure 1**). Thus, both time-dependent and tissue localization-dependent contexts may simultaneously regulate the role and effect of eATP receptors for T cell responses, although this has not been specifically studied.

## 5. eATP signaling as a T cell therapeutic target

Purinergic signaling is the target of many therapeutic studies that seek to balance the dual effect of eATP in infectious diseases, autoimmunity, and cancer. In severe tuberculosis and influenza, for example, sensing eATP via P2RX7-expressing CD4^+^ T cells increases disease severity. In this context, eATP signaling antagonism became the target of studies to prevent severe cases ^[67,68]^. Similarly, in cancers such as melanoma, neuroblastoma, and colon cancer, eATP signaling pharmacological blockade reduces vascular endothelial growth factor (VEGF)-mediated tumor growth ^[69]^. On the other hand, some therapeutic strategies to stimulate eATP receptors had been tested to reduce tumor migration and growth ^[70]^, and to enhance the immune response against bacteria and parasites ^[37,71–73]^. Although many therapeutic strategies focused on eATP signaling are emerging, there is still a limitation of therapies specifically focused on T cells. The use of purinergic receptor antagonists or agonists faces the problem of the effects that eATP signaling has on different cell types. These approaches, nevertheless, had generated promising results that, if improved, could lead to efficient therapies to ameliorate disease.

## 6. Concluding remarks

In this mini-review, we revisited recent findings on how eATP receptors regulate conventional T-cell responses to disease. In summary, T cells use recognition of eATP via distinct receptors to regulate their activation, differentiation into effector and memory subsets, and survival or death. These contrasting fates are induced, in T cells, depending on two major extrinsic factors: tissue localization and time after activation. Those extrinsic factors may simultaneously influence the outcome of eATP sensing responses (**Figure 1**). Future, detailed investigations of how these factors are regulated by and modulate T cell populations will allow a better understanding of how eATP works as a microenvironmental driver of T cell function.

## Author contributions

H.B.d.S. conceptualized and wrote the review. I.S-C. and A.B. wrote the review.

## Conflicts of interest

H.B.d.S. is a Consultant for International Genomics Consortium. The other authors declare they have no conflicts of interest.

## Acknowledgments

We thank the Borges da Silva lab for intellectual input. H.BdS. is funded by the National Institutes of Health, National Institute of Allergy and Infectious Diseases (1R01AI170649-01).
